# Mr. Pope, on Dr. Kinglake's Treatment of Gout

**Published:** 1807-07

**Authors:** T. Pope

**Affiliations:** Cleobury, Salop


### *To the Editors of the Medical and Physical Journal.*

GENTLEMEN,

THE two subjoined Cases, I presume, may not be unacceptable to the generality of the readers of your highly useful Journal.

Having had three instances of the bad effects of Dr. Kinglake's cooling treatment of the Gout, I have selected the following from among them, as it is the only one of which I took notes at the time. I do not bring it forward from any disrespect to Dr. K. but for the good of our fellow-creatures: and I will do Dr. K. the justice, to allow that that he is actuated by the same laudable motive; and that it will have its weight in inducing him to renounce a practice often fraught with the most mischievous and dreadful consequences; for he has repeatedly declared that he is ever open to conviction.

I am ready to admit, that suddenly cooling the extremities in gout, has, like a charm, had the most instantaneous and pleasing effects; and I am as ready to admit, that in many instances it has been productive of metastases, producing death, and very often states nearly bordering on it. Let us not in this enlightened age, give up that knowledge we have of the animal œconomy, and instead of employing safe and sure means, make use of those almost approaching to empiricism.

Some one lately advanced, that the gouty inflammation requires the same treatment as other inflammations. This then is equivalent to saying, that the gouty inflammation is unconnected with the general constitution; for we know that many inflammations are merely topical, requiring only a local treatment; whereas the cause of gouty inflammation is, in my opinion, something generated in the constitution by the mode of living; excess of acids very much conduces to it. The gout and gravel have a near affinity with each other, and are most commonly, I had nearly said always, concomitants.

Not being disposed at present to prosecute this subject farther, and fearing lest I have taken up too much of your valuable room, I shall proceed to the cases, the latter of which I shall introduce without any comment.

I am, &c.

*Cleobury, Salop, May* 29, 1807. T. POPE.

---

Case I. On the 7th of last July, I was desired to visit Mrs. ———, an inhabitant of this place, whom I found labouring under a most agonizing pain of the stomach, attended with nausea, vertigo, and a pulse not exceeding 54 strokes in a minute. She had been in this condition three hours before I saw her. On inquiring into the cause of these alarming symptoms, I was informed that three days before, as she was coming down stairs her left foot slipped, which occasioned a trifling sprain of the ancle; trifling, I say, because on the following morning she felt little inconvenience from it; however, in the evening of the same day, a violent pain with considerable inflammation, laid hold of it; so much so as to render her incapable ble of walking. On the morning of the 6th, she applied to the ancle and foot (for the great toe now likewise participated) by the advice of a neighbour, rags wetted with cold water, mixed with a small quantity of Goulard's extract. This almost immediately gave relief, and before night she could walk about the room. But this short-lived ease was dearly bought, for in the morning the above-mentioned symptoms supervened. I should have observed, that she was about forty-eight years old, of a strong muscular make, and florid complexion; paternally inheriting the gout, an attack of which she was accustomed to have, for the last twenty years, every two or three years. Reasonably alarmed for her safety, I employed the strongest stimuli, together with immersing her foot for five minutes every now and then in warm water, and in the intermediate time enclosing it in flannel; yet, until the 9th, without the least amendment, when the regularly morbific action resumed its salutary course, run its accustomed race, and left her as it was used to do.

Case II. On the 8th of last June, I was sent for to a respectable farmer in this neighbourhood, ætatis 50. On my arrival, I found him labouring under a most harrassing and incessant cough, pain in the upper part of the sternum, an excessive discharge of thin mucus, and pulse 130. Inquiring into the time of the first appearance and cause of these symptoms, I was informed that three days before he was taking pease-pottage, when the cough suddenly seized him, and that it was supposed to be further aggravated by cold, as he went and seated himself at the door for about an hour. After receiving this statement, I had no doubt but that one of the peas had slided by the epiglottis, and descended into the bronchiæ; and on this conviction I acted. Finding, as I have before observed, the cough hardly allowing him a moment's interval of rest, the bronchial discharge thin, excessive, and a little tinged with blood, and the symptomatic fever alarmingly high, I thought my patient, for obvious reasons, in the most dangerous situation; however, by a large bleeding, sedative and mucilaginous medicines, and a cooling diet, I had the satisfaction, in the course of a few days, to enable him to lie down in bed, and sleep six hours without intermission. In a few days more he took up the pike, and exercised it in the hay-field. But all was not yet over, though he thought himself in good health; for he had a little cough, and I was not convinced that the cause of the the late dreadful ills was removed. He continued in this manner until the 3d of August, when the cough suddenly rose to its originally alarming height, and after the most violent efforts, expelled the offending pea on the following morning; since when the cough gradually declined, has been three or four months entirely gone, and left my patient in perfect health.

